# Exploiting within‐breed variability in the autochthonous Reggiana breed identified several candidate genes affecting pigmentation‐related traits, stature and udder defects in cattle

**DOI:** 10.1111/age.13109

**Published:** 2021-06-28

**Authors:** S. Bovo, G. Schiavo, H. Kazemi, G. Moscatelli, A. Ribani, M. Ballan, M. Bonacini, M. Prandi, S. Dall’Olio, L. Fontanesi

**Affiliations:** ^1^ Division of Animal Sciences Department of Agricultural and Food Science University of Bologna Viale Giuseppe Fanin 46 Bologna 40127 Italy; ^2^ Associazione Nazionale Allevatori Bovini di Razza Reggiana (ANABORARE) Via Masaccio 11 Reggio Emilia 42124 Italy

**Keywords:** *Bos taurus*, bovine, coat colour, morphometry, muzzle, SNP, supernumerary teats, teat length

## Abstract

Autochthonous cattle breeds constitute important reservoirs of genetic diversity. Reggiana is an Italian local cattle breed reared in the north of Italy for the production of a mono‐breed Parmigiano–Reggiano cheese. Reggiana cattle usually have a classical solid red coat colour and pale muzzle. As part of the strategies designed for the sustainable conservation of this genetic resource, we investigated at the genome‐wise level the within‐breed detected variability of three pigmentation‐related traits (intensity of red coat colour, based on three classes – light/diluted, normal and dark; spotted patterns/piebaldism that sometime emerge in the breed; muzzle colour – pink/pale, grey and black), stature, presence/absence and number of supernumerary teats and teat length. A total of 1776 Reggiana cattle (about two‐thirds of the extant breed population) were genotyped with the GeneSeek GGP Bovine 150k SNP array and single‐marker and haplotype‐based GWASs were carried out. The results indicated that two main groups of genetic factors affect the intensity of red coat colour: darkening genes (including *EDN3* and a few other genes) and diluting genes (including *PMEL* and a few other genes). Muzzle colour was mainly determined by *MC1R* gene markers. Piebaldism was mainly associated with *KIT* gene markers. Stature was associated with BTA6 markers upstream of the *NCAPG*–*LCORL* genes. Teat defects were associated with *TBX3*/*TBX5*, *MCC* and *LGR5* genes. Overall, the identified genomic regions not only can be directly used in selection plans in the Reggiana breed, but also contribute to clarifying the genetic mechanisms involved in determining exterior traits in cattle.

## Introduction

Autochthonous breeds, available in all main livestock species, constitute important reservoirs of genetic diversity. These breeds are, in general, well adapted to the production systems in which they have been developed. Breeds can usually be distinguished by associated inheritable exterior phenotypes that could be considered in many cases breed‐specific traits and, for that reason, they might be fixed or close to the fixation in the breed population. Some degrees of within‐population variability could, however, exist for some of these descriptors, mainly in local breeds, owing to recent admixture with other populations or relaxed selection pressures towards standard forms, or because some degrees of variability are allowed in the population. As such, within‐breed phenotypic variability can be useful to identify markers associated with different expression of exterior features that could help, in turn, to understand the genetic architecture of these traits and then to apply genomic information to facilitate the fixation of standardised phenotypes in the targeted breeds (Schiavo *et al*. 2018, 2019).

Reggiana is an Italian autochthonous cattle breed, reared mainly in the Emilia Romagna region (the north of Italy), with a high density of farms that raise this breed in the province of Reggio Emilia (from which its name comes), in the well‐known worldwide Protected Designation of Origin Parmigiano–Reggiano cheese production area. Milk of this breed is almost exclusively utilised to produce a mono‐breed Parmigiano–Reggiano cheese, that is labelled with a brand name (*Vacche Rosse*) created to refer to the characteristic typical red coat colour of the Reggiana cattle (known as *fromentino*; Russo *et al*. 2007). The creation of this mono‐breed branded Parmigiano–Reggiano cheese in the 1990s reverted the progressive decline of the Reggiana population that had occurred over the previous decades, reaching a minimum number of about 500 cows in the 1980s. The premium price that this branded Parmigiano–Reggiano cheese can obtain, if compared with undifferentiated Parmigiano–Reggiano cheese, has ensured an economic remuneration to the farmers that compensates for the lower productivity of the Reggiana cows in comparison with that of other cosmopolitan dairy breeds (Gandini *et al*. 2007; Fontanesi 2009). The resulting economic advantage for Reggiana cattle farmers made it possible to support a successful conservation programme for this genetic resource strictly interconnected with its cheese. The current Reggiana cattle population comprises about 2800 cows registered to its breed Herd Book, distributed over about 100 farms (ANABORARE 2020).

A few studies have been carried out to characterise this breed at the genetic level. Mariani & Russo (1971) first investigated the frequency distribution of k‐casein protein variants in the Reggiana population. Then, Caroli *et al*. (2004) monitored the presence of polymorphisms in three caseins and in *β*‐lactoglobulin by isoelectrofocusing on the milk of Reggiana cows. SNPs in several candidate genes affecting milk production traits have been subsequently reported (Fontanesi *et al*. 2015). Coat colour genes have also been investigated in Reggiana cattle (Russo *et al*. 2007; Fontanesi *et al*. 2010a, 2010b, 2012). The genetics of the basic coat colour in this breed is well established: the recessive allele at the *Extension* locus/melanocortin 1 receptor (*MC1R*) gene (allele *e*) determines the classical red coat colour (*fromentino*) of this breed (Klungland *et al*. 1995; Russo *et al*. 2007). Coat colour has been the only exterior trait that has been investigated using molecular markers in this breed. Other conformation traits or udder‐related traits that might be relevant to explain performances and potentials of this breed have not been explored yet.

Genome‐wide analyses using SNP arrays have been conducted in Reggiana cattle to identify breed‐informative markers that could distinguish Reggiana animals from those of other cosmopolitan breeds (Bertolini *et al*. 2015, 2018) and to compare the distribution of ROHs in this breed and in other cosmopolitan and local breeds (Mastrangelo *et al*. 2016, 2018a). Two other studies have evaluated the genetic relationships between Reggiana and several other Italian breeds and compared selection signatures across breeds (Mastrangelo *et al*. 2018b; Bertolini *et al*. 2020). The Reggiana cattle genome was shown to still contain several signatures that are reminiscent of its past un‐specialised purpose (Reggiana was a triple‐purpose breed, dairy–beef–work, until the 1960s), before it was redirected towards a dairy specialisation (Bertolini *et al*. 2020), partially through some early and mostly undeclared introgression events from other more productive dairy breeds. Therefore, despite the quite high pressure to maintain the Herd Book breed standard for exterior traits (solid *fromentino* coat colour, pink/pale muzzle, medium‐tall stature and a dairy or dual‐purpose conformation), some Reggiana animals not fully matching the classical exterior descriptors of the breed can emerge sometimes over generations and some phenotypic variability is present in the population. Therefore, a phenotyping programme embracing a large fraction of the breed population has been established, with the aim of monitoring the level of compliance of the Reggiana population to the Herd Book standard and subsequently planning actions to introduce breed‐specific exterior features in the selection goals of this population and reduce the frequency of morphological defects. The programme is part of the strategies that have been designed for the sustainable conservation of this genetic resource and it is also planned to use genetic markers to achieve its aims.

In this study, we took advantage of the phenotyping data collected in the Reggiana breed and investigated at the genome‐wide level the association of SNPs with several exterior traits that are relevant for maintaining breed‐specific features and for their detailed characterisation (intensity of red coat colour, muzzle colour and coat colour patterns), for the description of the body structure and related purpose of the animals (stature) and teat‐related traits that are directly or indirectly involved in udder health and milking adaptability (supernumerary teats and teat length). The results identified new candidate genes and confirmed the role of some additional genes that have been already indicated to affect the same or similar traits. The obtained information not only can be directly used in selection plans in the Reggiana breed but also contributes to clarifying the genetic mechanisms involved in determining exterior traits in cattle, including novel aspects related to pigmentation in *Bos taurus*.

## Materials and methods

### Animals and phenotypes

**Figure 1:**
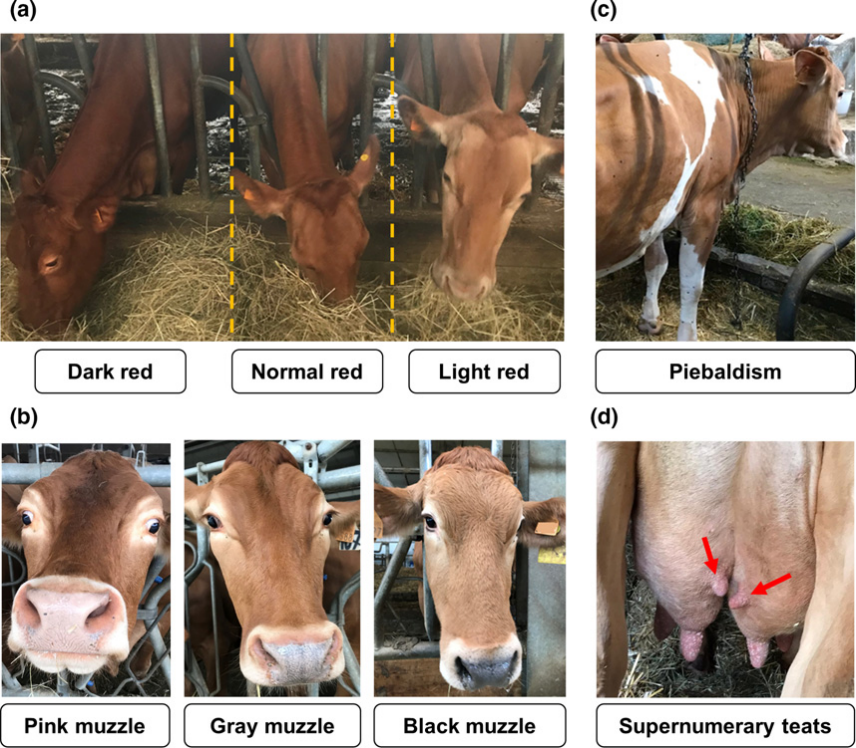
Examples of the exterior traits investigated in Reggiana cattle: (a) different grades of red coat colour (dark, normal, and light/diluted red); (b) different muzzle colour classes (pink/pale, grey and black); (c) piebaldism/spotted; and (d) supernumerary teats.

A total of 1796 Reggiana cattle, born over the years 2000–2019, were included in this study. The exterior traits of animals, recorded by trained personnel, were the following (Table 1): (i) intensity of red coat colour based on three classes – light or diluted red (L), normal red (N) and dark red (D) (Fig. 1a); (ii) muzzle colour based on three classes – pink or pale (P), grey (G) and black (B) (Fig. 1b); (iii) coat colour distribution based on two classes – uniform (solid red) and non‐uniform (red with white spots, indicated as piebaldism; Fig. 1c); (iv) stature of the animals measured at the hip (cm); (v) the presence and the number of supernumerary teats (Fig. 1d); and (vi) teat length (averaged over the normal four teats (cm). Not all animals that were phenotyped had information for all of the mentioned traits (Table 1).

**Table 1:** Details of the GWASs for three pigmentation‐related traits (intensity of red coat colour, muzzle colour and piebaldism/spotted patterns), stature and two udder‐related defects (supernumerary teats and teat length) that were carried out in Reggiana cattle.

Trait	GWAS approach^1^	Number of animals	*λ*_GC_ ^2^	*h* ^2^ (SE)^3^	*λ*_GC_ ^4^	*h* ^2^ (SE)^5^
Intensity of red coat colour^6^	Continuous variable	1213	1.030	0.34 (0.05)	1.015	0.34 (0.05)
	Light vs. normal	1083	1.016	0.09 (0.05)	0.998	0.08 (0.04)
	Light vs. dark	198	1.016	0.80 (0.16)	1.003	0.82 (0.16)
	Normal vs. dark	1145	1.017	0.34 (0.05)	1.018	0.33 (0.05)
	Light vs. normal + dark	1213	1.008	0.10 (0.04)	0.988	0.10 (0.04)
	Light + normal vs. dark	1213	1.020	0.34 (0.05)	1.018	0.34 (0.05)
Muzzle colour^7^	Continuous variable	1014	1.003	0.38 (0.06)	1.008	0.37 (0.06)
	Pink vs. grey	951	1.012	0.20 (0.06)	1.012	0.20 (0.06)
	Pink vs. black	848	1.025	0.52 (0.07)	1.026	0.49 (0.08)
	Grey vs. black	229	1.027	0.60 (0.14)	1.030	0.60 (0.14)
	Pink vs. grey + black	1014	1.011	0.28 (0.12)	1.014	0.27 (0.12)
	Pink + grey vs. black	1014	1.024	0.46 (0.07)	1.024	0.41 (0.07)
Piebaldism^8^	Solid coloured vs. spotted	1288	1.031	0.53 (0.07)	1.016	0.45 (0.08)
Stature (hip height)^9^	Continuous variable	1279	0.999	0.34 (0.06)	1.015	0.30 (0.05)
Supernumerary teats^10^	Presence vs. absence	1112	1.004	0.25 (0.05)	1.003	0.25 (0.05)
	Continuous variable	1112	0.999	0.29 (0.05)	0.994	0.30 (0.06)
Teat length^11^	Continuous variable	1258	1.002	0.35 (0.05)	1.001	0.36 (0.06)

^1^ Covariates used in the linear mixed model were the following: technician who recorded the trait in all models; month of recording in all models that investigated the intensity of coat colour; age of the animals for stature and teat length. Several other factors were tested but were not included because were not significant.

^2^ Genomic control inflation factor (single‐SNP genome scan).

^3^ Genomic heritability estimated from SNP data (single‐SNP genome scan). The standard error is reported in parentheses.

^4^ Genomic control inflation factor (haplotype‐based genome scan).

^5^ Genomic heritability estimated from SNP data (haplotype‐based genome scan). The standard error is reported in parentheses.

^6^ Animals presented the following red grades: light/pale red (*n* = 69, animal numerically coded as 1), normal red (*n* = 1041, animal numerically coded as 2) and dark red (*n* = 133, animal numerically coded as 3). The GWAS approach ‘Continuous variable’ evaluated the trait in a quantitative way. The other remaining genome scans were addressed as case–control studies (traits coded in a binary form, 0 and 1).

^7^ Animals presented the following muzzle colour: pink (*n* = 785; animal coded as 0), grey (*n* = 166, animal coded as 1) and black (*n* = 63, animal coded as 2). The GWAS approach ‘Continuous variable’ evaluated the traits in a quantitative way. The other remaining genome scan were addressed as case–control studies (traits coded in a binary form, 0 and 1).

^8^ Case–control study. Trait coded in a binary form (0 and 1). Animals presenting a solid coat colour (*n* = 1232, animal coded as 0) were compared against animals with spotted patterns (*n* = 56, animal coded as 1).

^9^ Data treated in a quantitative way. The trait was recoded in cm. Minimum = 130 cm; maximum = 156 cm; mean = 141 cm; standard deviation = 4 cm; median = 142 cm. Age was measured in days (from 594 to 7537 days).

^10^ Animals presented the following supernumerary teats: 0 (*n* = 764 animals), 1 (*n* = 206 animals), 2 (*n* = 140 animals) and 3 (*n* = 2 animals). The GWAS approach ‘Presence vs. absence’ compared animals having a regular number of teats equal to four against animals having extra teats (case–control study; trait coded in a binary form, 0 and 1). The ‘Continuous variable’ approach analysed the trait in a quantitative way.

^11^ Data treated in a quantitative way. The trait was recoded in cm. Minimum = 2 cm; maximum = 9 cm; mean = 4.9 cm; standard deviation = 1.3 cm; median = 5 cm. Age was measured in days (from 594 to 7537 days).

### Genotyping data

Blood samples and buccal swabs were used for DNA with the Wizard Genomic DNA Purification kit (Promega Corporation). All animals were genotyped with the GeneSeek GGP Bovine 150k Array (which can analyse 139 458 SNPs). Standard genotyping protocols, based on the supplier’s recommendations, were used. plink 1.9 software (Chang *et al*. 2015) was used for quality checks. Animals with a genotyping call rate less than 0.95 were discarded. SNPs were discarded if they had call rate less than 0.90, a HWE *P* < 0.001 and a MAF less than or equal to 0.02. blast+ v.2.7.1 (Camacho *et al*. 2009) was used to map SNPs to the ARS‐UCD1.2 reference genome (GCA_002263795.2) and markers assigned to more than one position or assigned to sex chromosomes were discarded. The final datasets consisted of 1776 animals and 122 631 SNPs.

### Phasing and haplotype estimation

SNP data were phased using shapeit v.2 (Delaneau *et al*. 2011) using a window size of 2 Mb and an effective population size (*N*_e_) of 201 estimated with snep v.1.1 (Barbato *et al*. 2015). A genome window of 100 kb with a sliding block of 50 kb was used to estimate haplotypes in r (R Core Team 2020) using the package ghap 1.2.2 (Utsunomiya *et al*. 2016). This window size was selected based on an LD (*r* ^2^ value) analysis that pointed out a rapid decline of LD such that *r* ^2^ ≈ 0.1 at 100 kb. Haplotypes were treated as bi‐allelic markers and genotypes were coded as NN, NH and HH genotypes (H = haplotype allele and N = NULL = all other *N* alleles) in the subsequent analyses. A regular ped file was obtained with plink 1.9 software, filtering out haplotypes presenting a MAF less than or equal to 0.02. The final datasets consisted of a total of 1776 animals and 403 307 haplotypes and 49 994 haploblocks. Each block contained an average number of haplotypes (±standard deviation) equal to 8 ± 4 (min = 2, max = 29, median = 4). Haplotypes included an average number of SNPs equal to 5 ± 3 (min = 2, max = 53, median = 5). This was expected considering the genome size and the DNA marker distribution across the genome. The haplotype structure of regions of interest was further evaluated using haploview software (Barrett *et al*. 2005).

### GWASs with single markers and haplotypes

To dissect the genetic architecture affecting the recorded traits, GWASs were carried out including distinct groups of cattle as reported in Table 1. Association analyses were performed considering a linear mixed model that assumed a trend per copy of the minor allele that specifies the dependency of the phenotype on genotype categories. The following linear mixed effect model was used: y=Wα+xβ+g+e, where ***y*** (*n* × 1) is a vector containing the phenotype for the *n* ^th^ animal, ***W*** (*n* × *k*) is a covariate matrix and ***α*** is the *k*‐dimensional vector of covariates effects, ***x*** (*n* × 1) is the vector containing genotypes for the *i* ^th^ DNA marker, *β* is the additive fixed effect of the *i* ^th^ DNA marker (SNP or haplotype), $g\approx N(0,\sigma_{g}^{2}K)$ is a multivariate Gaussian polygenic effect, with covariance matrix proportional to the relatedness matrix **K** (*n* × *n*), and $e\approx N(0,\sigma_{e}^{2}I)$ is a multivariate Gaussian vector of uncorrelated residuals. The assessment of the association between each DNA marker/haplotype and the targeted phenotype was obtained by testing the null hypothesis *H*_0_:*β* = 0. Significance was obtained using the Wald test. All of the models were fitted with gemma v.0.98 (Zhou & Stephens 2012) after computing the relatedness matrix **K** as a centred genomic matrix. Two **K** matrices were produced: one from SNPs and another from haplotypes. Details about the linear mixed effect models are reported in Table 1. In some models the analysed phenotypes were considered as a continuous variable and in other models, where the phenotype was registered in three phenotypic classes (i.e. intensity of red and muzzle colour), two groups were defined based on biological assumptions and case–control studies were run (Table 1). The top associated SNP was further included in the model as fixed effect to confirm association and to identify other relevant genomic regions that could eventually emerge. The Bonferroni‐corrected significance level threshold equal to a nominal value of 0.05 was used to define significant markers and haplotypes. A threshold of *P* = 5.0 × 10^−6^ was applied to identify suggestively associated markers or haplotypes. gemma was used to estimate the genomic (chip) heritability ($h_{G}^{2}$). Genomic control inflation factors (*λ*_GC_) were computed in r. QQ and Manhattan plots were generated in r using the *qqman* package (Turner 2018).

Protein coding genes annotated in the ARS‐UCD1.2 genome version spanning the significantly associated haplotypes and a region of ±500 kb around the significantly associated SNPs were retrieved from the related NCBI GFF file using bedtools v.2.17.0 (Quinlan & Hall 2010). Manual annotation and a literature search were applied to identify candidate genes that could affect the analysed traits.

## Results

### Phenotypic characterisation of Reggiana cattle

Phenotyping of the investigated cattle was obtained for three pigmentation‐related traits (Fig. 1a–c). Considering the intensity of red colour of the coat of Reggiana cattle, three main grades of red could be easily detected (Fig. 1a). Reggiana cattle were usually characterised by a normal red coat colour (83.7% of the phenotyped animals), which is classically referred as *fromentino*, whereas a smaller fraction of the breed population had light/diluted red (5.6%) or a dark red coat colour (10.7%). Another pigmentation‐related trait was defined according to the three observed muzzle colour classes indicated in Fig. 1b. Most of the animals had a pink/pale muzzle (77.4%), which is the characteristic feature of the breed. Grey and black muzzle classes were reported for 16.4 and 6.2% of cattle respectively. The third pigmentation‐related trait considered the presence or absence of piebaldism. Spotted patterns (of any type) were observed in only 4.3% of cattle (Fig. 1c). The remaining 95.7% of the animals had the classical solid red coat colour (of one of the three grades described above).

Stature of the animals was in the range 130–156 cm (mean ± SD = 141 ± 4 cm, median = 142 cm). All animals had at least four nipples and about 31% of the cattle had one (*n*. 206, 18.5%), two (*n*. 140, 12.5%) or three (*n*. 2, <1%) supernumerary teats (Fig. 1d). Teat length ranged from 2.0 to 9.0 cm (mean ± SD = 4.9 ± 1.3 cm, median = 5.0 cm).

### Markers and haplotypes associated with several phenotypes

**Figure 2:**
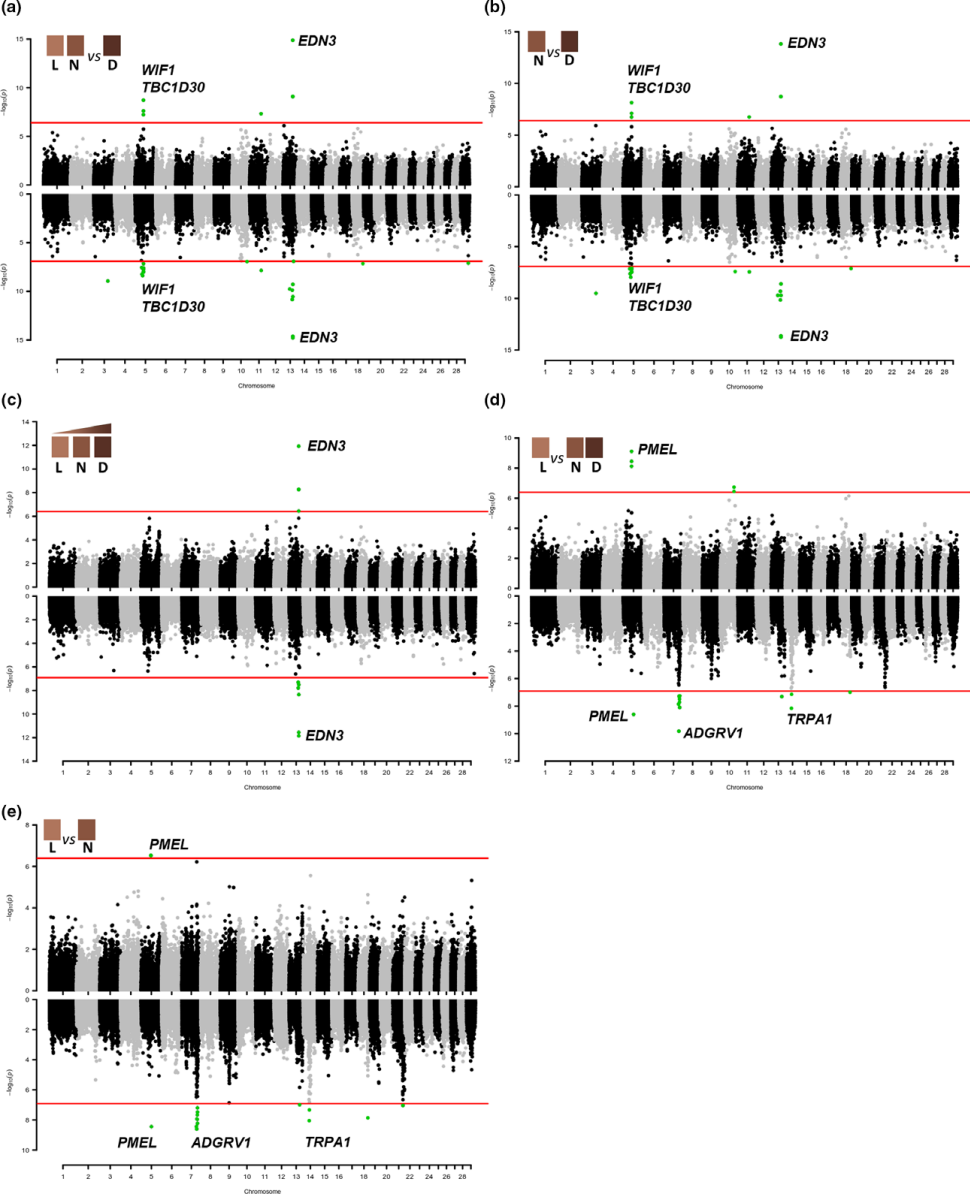
Manhattan plots of the GWASs for the intensity of the red coat colour using the three classes of red grades [light/diluted (L), normal (N) and dark (D)]: (a) L plus N vs. D; (b) N vs. D; (c) continuous model with the three classes; (d) L vs. N plus D; (e) L vs. N. The results of the single‐marker analysis are on the top part of each plot and the results of the haplotype‐based analysis are on the bottom part of each plot. Each dot represents an SNP or a haplotype. The red line identifies the significance threshold (Bonferroni correction; *α* = 0.05).

**Figure 3:**
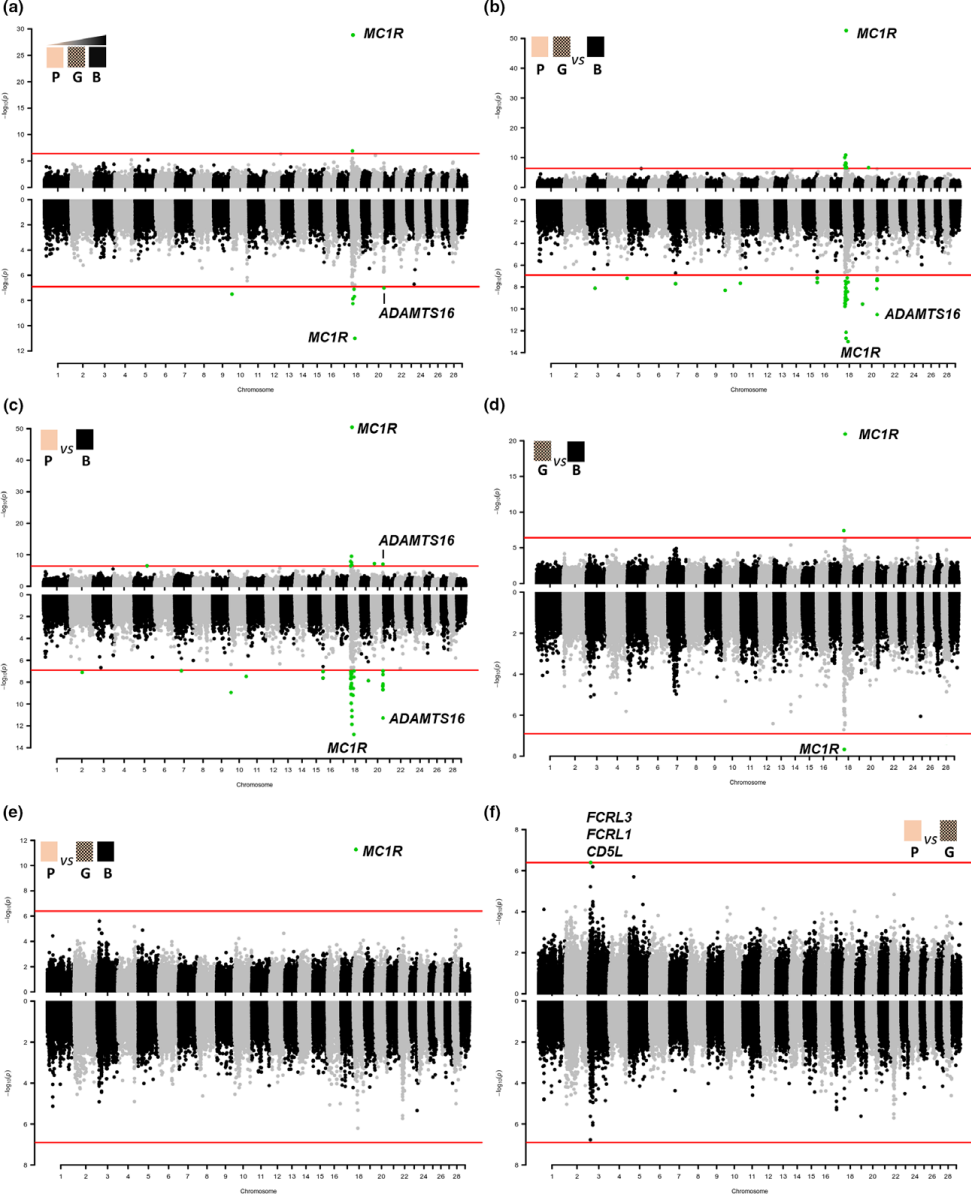
Manhattan plots of the GWASs for the muzzle colour [pink/pale (P), grey (G) and black (B)]: (a) muzzle colours as continuous variable (from pink to black); (b) P plus G vs. B; (c) P vs. B; (d) G vs. B; (e) P vs. G plus B; (f) P vs. G. The results of the single‐marker analysis are on the top part of each plot and the results of the haplotype‐based analysis are on the bottom part of each plot. Each dot represents an SNP or a haplotype. The red line identifies the significance threshold (Bonferroni correction; *α* = 0.05).

**Figure 4:**
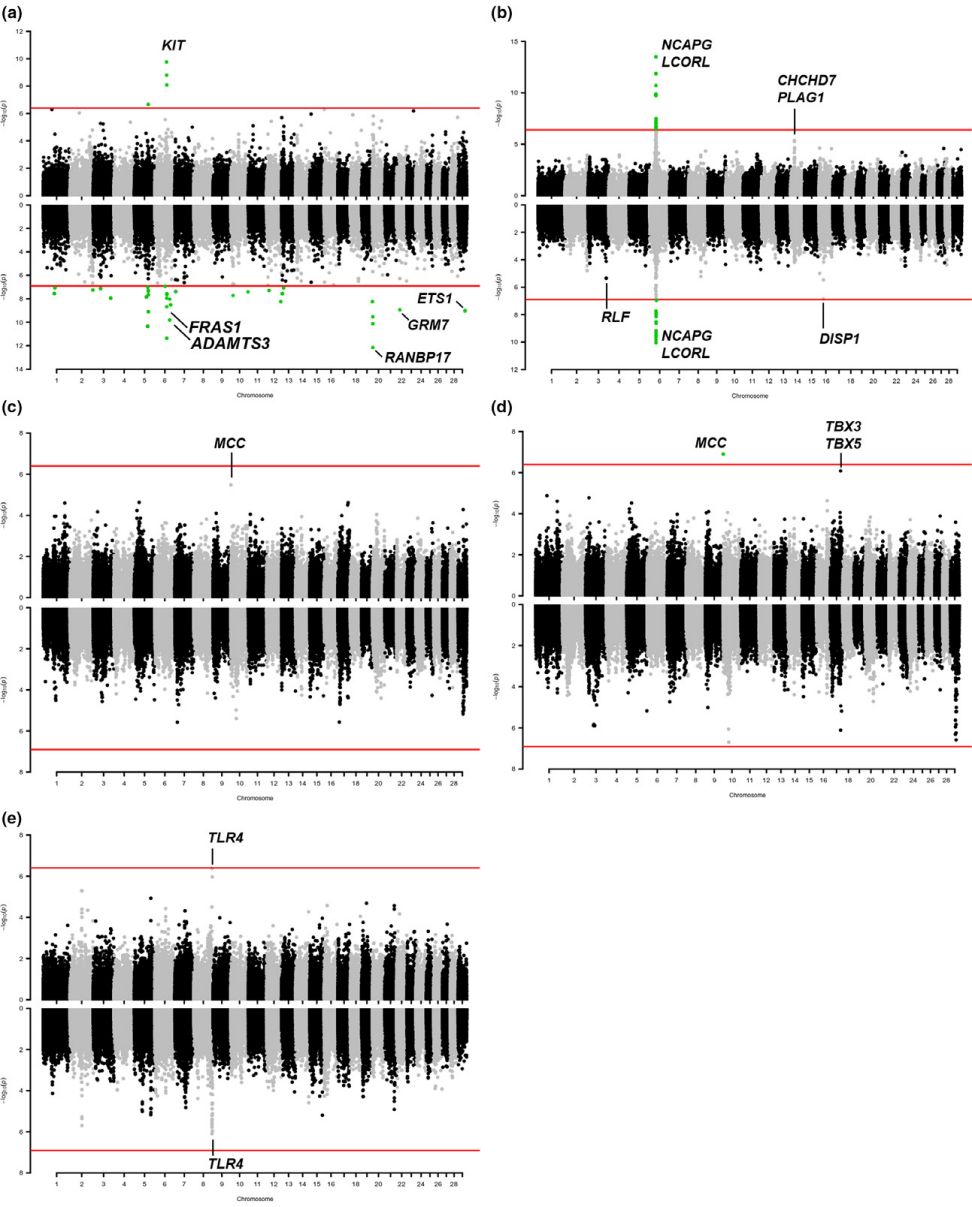
Manhattan plots of the GWASs in Reggiana cattle for (a) piebaldism/spotted – solid coloured class vs. spotted class; (b) stature; (c) presence/absence of supernumerary teats; (d) number of supernumerary teats; and (e) teat length. The results of the single‐marker analysis are on the top part of each plot and the results of the haplotype‐based analysis are on the bottom part of each plot. Each dot represents an SNP or a haplotype. The red line identifies the significance threshold (Bonferroni correction; *α* = 0.05).

Data were treated with different models as described in Table 1. Genomic control inflation factor values were all close to 1 (Table 1), indicating that population stratification was correctly accounted for in all analyses. Table 2 reports the most relevant associated genomic regions identified by the single‐marker and haplotype‐based GWASs whereas the full set of significant and suggestively significant associations (*P* < 5.0 × 10^−6^) is reported in Tables S1 (single‐marker) and S2 (haplotype‐based). Manhattan plots showing the results of the different GWASs for the intensity of red are reported in Fig. 2. Figure 3 reports the Manhattan plots obtained for the GWASs that were used to dissect the muzzle colour diversity. Figure 4 includes the Manhattan plots derived by the analyses for piebaldism that sometimes occurred in the breed and for the morphometric features (stature and teat‐related traits). QQ plots are available in Figs S1 and S2 for single‐marker and haplotype‐based genome scans, respectively.

**Table 2:** Genomic regions harbouring candidate genes affecting morphological traits and defects investigated in the Reggiana cattle breed.

Trait	Comparison(s)	BTA:position^1^	Gene(s)	SNPs^2^	Haplotypes^2^
Intensity of coat colour (red grades)	Continuous variable; light + normal vs. dark; normal vs. dark	13:57.03	*EDN3*	Associated	Associated
	Normal vs. dark; light + normal vs. dark	5:48.57	*LEMD3*, *WIF1*, *TBC1D30*	Associated	Associated
	Light vs. normal + dark; light vs. normal	5:57.34	*PMEL*	Associated	Associated
		7:86.4–91.2	*ADGRV1*	Suggestive	Associated
		14: 34.60–35.60	*TRPA1*	–	Associated
Muzzle colour	Continuous variable; pink + grey vs. black; grey vs. black; pink vs. grey + black; pink vs. black	18:14.70	*MC1R*	Associated	Associated
	Pink vs. black	20:67.30–67.90	*ADAMTS16*	Associated	Associated
	Continuous variable; pink + grey vs. black	20:67.30–67.90	*ADAMTS16*	Suggestive	Associated
	Pink vs. grey	3:12.67	*FCRL3*, *FCRL1*, *CD5L*	Associated	Suggestive
Piebaldism	Solid coloured vs. spotted	6:70.05–70.15	*KIT*	Associated	Associated
		6:87.35–87.50	*ADAMTS3*	–	Associated
		6:93.05–93.15	*FRAS1*	–	Associated
		20:2.6–2.9	*RANBP17*	–	Associated
		22:19.45–19.55	*GRM7*	–	Associated
		29:31.9–32.0	*ETS1*	–	Associated
Stature	Continuous variable	6:37.10‐38.00	*FAM184B*, *NCAPG*, *LCORL*, *LAP3*	Associated	Associated
		14:23.30	*PLAG1*, *CHCHD7*	Suggestive	–
		16:26.20–26.35	*DISP1*	–	Suggestive
		3:105.85–106.00	*RLF*	–	Suggestive
Supernumerary teats (ST)	Absence vs. presence of ST	10:0.89	*MCC*	Suggestive	–
	Number of ST	10:0.89	*MCC*	Associated	–
		17:60.4	*TBX3*, *TBX5*	Suggestive	Suggestive
Teat length	Continuous variable	8:106.95‐107.55	*TLR4*	Suggestive	Suggestive

Only the most relevant results of the GWASs detailed in Table 1 have been reported. The full set of associations is given in Tables S1 & S2 for single‐marker and haplotype‐based genome scans respectively.

^1^ Position of the most significant SNP or haplotype in basepairs on the *Bos taurus* chromosome (reference genome, ARS‐UCD1.2).

^2^ Associated (*P*_Bonferroni_ < 0.05) or suggestive (*P* < 5.00 × 10^−6^). The symbol ‘–’ denotes that any interesting marker is highlighted.

Estimated genomic heritability from single‐marker analysis (Table 1) ranged from 0.09 (for a comparison related to the intensity of red colour: light vs. normal) to 0.80 (for another comparison related to the intensity of red colour: light vs. dark). These extreme values were also observed with the haplotype‐based estimates of heritability (Table 1).

#### Intensity of red coat colour

The GWASs that included three different classes of red coat colour grades and different comparisons were designed to potentially dissect the genetic architecture affecting the colour intensity. The obtained results indicated that there might be two main general groups of factors underlying this trait: some factors that darken the basic red colour (which emerge or that are confirmed when the dark red class is contrasted with the normal red class or both the light and normal classes) and some others that might lighten or dilute the regular red grade (which emerge or are confirmed when the light red class is contrasted with the normal red class or with the normal red class together with the dark red class).

We first report the results that could explain the mechanisms that might be involved in the darkening of the red, which also include the most significant associations obtained in all analyses related to the intensity of the red colour. Both GWASs carried out using single‐markers and haplotypes returned a major peak of association in the same chromosome region of BTA13 (Table 2): (i) when light red and normal red were grouped together against dark red in a case–control study (Fig. 2a) and (ii) when only the normal red class was analysed against the dark red class in a case–control comparison (Fig. 2b). The most significant SNP in the two single‐marker models was ARS‐BFGL‐NGS‐27559 (rs41694067) with *P* = 1.32 × 10^−15^ and *P* = 1.52 × 10^−14^ respectively (Table S1). These results were also confirmed when the three phenotypic classes (L, N and D red) were included separately in the model and treated as a quantitative trait (Fig. 2c), even if less significant (*P* of the same SNP was equal to 1.19 × 10^−12^). This marker is located at nucleotide position 57037937, close to the region of the *endothelin 3* (*EDN3*) gene (ENSBTAG00000012109; annotated from nucleotides 57061094–57088977). The two most significant haplotype windows in all three GWASs (Table S2) perfectly overlapped this gene. It is well established that *EDN3* is essential in the development of epidermal melanocytes and mediates an increase in melanocyte density (Baynash *et al*. 1994; Lahav *et al*. 1996, 1998; Reid *et al*. 1996; Opdecamp *et al*. 1998).

Another peak of association was obtained on BTA5 (around 48.57 Mb; Fig. 2a,b) where single‐marker and haplotype‐based association studies that compared (i) the light red plus normal red classes grouped together against the dark red class in a case–control model and (ii) when only the normal red class was analysed against the dark red class in a case–control comparison evidenced MS‐rs209627948 (nucleotide position 48572519) as the most significant SNP in this region (*P* = 1.90 × 10^−9^ and 7.10 × 10^−9^ for the two analyses respectively). This SNP is a splice region variant in the *LEM domain containing 3* (*LEMD3*) gene, whose known functions are not directly related to pigmentation. However, two other genes located in this region [*WNT inhibitory factor 1* (*WIF1*) gene (positions: 48687578–48779884 bp) and *TBC1 domain family member 30* (*TBC1D30*) gene (positions: 48952181–49035846 bp)], which encode for factors involved in pigmentation processes, have been associated with hair colour in humans (Kichaev *et al*. 2019).

Another significant marker that emerged in the GWASs that compared the same two groups of red intensity classes was identified on BTA11 (ARS‐BFGL‐BAC‐14940; rs110968529 at position 66025832 bp). The results were confirmed by the haplotype‐based analyses (Tables S1 & S2). None of the genes in this region have been described as being involved in pigmentation. For the same association analyses, a few additional significant haplotypes (Table S2) were identified on BTA3 (81.25–81.35 Mb; confirmed by a suggestively significant marker; Table S1), BTA5 (37.85–39.80 Mb, 43.45–43.55 Mb), BTA10 (86.90–87.00 Mb), BTA13 (61.40–61.50 Mb), BTA18 (61.55–61.65 Mb) and BTA29 (45.35–45.45 Mb).

Among the second group of results that highlighted coat colour dilution factors, the most significant polymorphism in the single‐marker analysis was obtained when (i) the light red class was compared with the other two classes grouped together (Fig. 2d) and when (ii) the light red class was contrasted only with the normal red class (Fig. 2e) was a variant in the *premelanosome protein* (*PMEL*, also indicated as *PMEL17* or *SILV*) gene (Tables 2 & S1; *P* = 6.72 × 10^−8^ and *P* = 2.98 × 10^−7^ respectively). This marker (rs385468954; 5:g.57345305_57345307del) is determined by a three‐nucleotide in‐frame deletion in exon 1 of *PMEL*, which eliminates the leucine residue at position 19 of the ENSBTAP00000005250.4 encoded protein sequence (p. Leu19del). This mutation has already been associated with a diluted coat colour phenotype in other cattle populations (Jolly *et al*. 2008; Schmutz & Dreger 2013). This genomic region was also confirmed by the haplotype‐based analyses (Tables 2 & S2). Other significant results that were derived from the comparison between the light class and the other two classes were obtained with the haplotype‐based approach. An additional relevant region obtained by this method, included several haplotype windows spanning from 86.4 to 91.2 Mb of BTA7 (Tables 2 & S2; Fig. 2d,e). Among the genes annotated in this region, the *adhesion G protein‐coupled receptor V1* (*ADGRV1*) gene has been reported to contribute to the variability of human hair colour (Morgan *et al*. 2018). Several other significant haplotypes identified with the same comparisons were also reported on BTA14 (34.60–35.60 Mb), where the transient receptor potential cation channel subfamily A member 1 (*TRPA1*) gene (positions 35611321–35666918 bp) is located, which encodes for an ion channel specifically expressed and acting in epidermal melanocytes (Corey *et al*. 2004). A few significant haplotype regions from the same GWASs were also reported on BTA13 (62.00–62.10 Mb), BTA18 (55.90–56.00 Mb) and BTA21 (59.45–59.55 Mb; Table S2).

#### Muzzle colour

Single marker analysis that was run considering separately all three phenotypic classes (pink/pale, grey and black muzzles) identified a peak of highly significant markers (*P* = 1.41 × 10^−29^) on BTA18 within the *melanocortin 1 receptor* (*MC1R*) gene (Table 2 and Fig. 3a; Table S1). The association with *MC1R* SNPs was further strengthened when pink and grey classes were combined in one group against the black muzzle class (*P* = 2.03 × 10^−53^; Fig. 3b) or when only the pink class was contrasted with the black class (*P* = 3.18 × 10^−51^; Fig. 3c) in case–control studies. When the grey muzzle class was compared with the black muzzle class or when the pink class was compared with the grey plus black classes the *MC1R* SNPs were again the most significant markers (*P* = 1.15 × 10^−21^ and *P* = 5.29 × 10^−12^ respectively; Table 2 and Fig. 3d,e; Table S1). Mutations in the *MC1R* gene (determining the allelic series at the *Extension* coat colour locus) have been already described to affect coat colour in many species, including cattle (Klungland *et al*. 1995). The top *MC1R* gene marker that was genotyped discriminates the frameshift mutation (which disrupts the MC1R protein and causes the recessive allele *e* at the *Extension* locus) from the other alleles at this gene (Klungland *et al*. 1995; Joerg *et al*. 1996). This mutation has also been shown to determine the classical red coat colour phenotype of the Reggiana cattle (Russo *et al*. 2007). The genotyping of this marker, however, indicated that allele *e* is not completely fixed in the Reggiana breed and other alleles that are associated with the coloured muzzle segregate in the population [allele frequencies: *f*(*e*) = 0.95, *f*(*E*) = 0.05; genotype frequencies: *f*(*ee*) = 0.905, *f*(*Ee*) = 0.090, *f*(*EE*) = 0.005; where *E* indicates all other alleles that are different from *e*]. The single marker GWASs with some models (pink plus grey vs. black; pink vs. black; continuous model) showed another significant SNP on BTA18 but at about 3 Mb upstream from the *MC1R* gene and the haplotype analysis identified a few additional significant haplotype windows that were just upstream *MC1R* (<1 Mb from it) or at about 10 Mb downstream (Table S2). It seems that these additional regions on BTA18 might be in high LD with the *MC1R* gene markers because, including the *MC1R* SNPs in the model as fixed effects, all of these additional significant signals on BTA18 disappeared.

Another significant region (Table 2 and Fig. 3) that was detected using different models with both single‐marker (pink vs. black muzzle classes) and haplotype‐based (continuous variable, pink vs. black classes, pink plus grey vs. black classes) approaches was on BTA20 (from 67.30 to 67.90 Mb). This chromosome region contains the *ADAM metallopeptidase with thrombospondin type 1 motif 16* (*ADAMTS16*) gene whose function has not been completely characterised yet. A few other genes of the ADAMTS family are however involved in pigmentation defects or in melanoblast survival (Rao *et al*. 2003; Silver *et al*. 2008).

Some other significant SNPs or haplotypes were identified by the models that contrasted the black muzzle class with the pink or the combined pink plus grey muzzle groups (in a few cases confirmed by the continuous variable model; Tables S1 & S2). These significant signals were located on BTA2 (69.95–70.05 Mb), BTA3 (44.05–44.15 Mb), BTA4 (111.65–111.75 Mb), BTA5 (a SNP at position 75683960 bp), BTA7 (39.55–39.65 Mb), BTA10 (36.50–37.50 and 92.95–93.05 Mb), BTA15 (82.80–82.90 Mb) and BTA19 (45.20–45.30 Mb). None of these regions contain obvious candidate genes whose role might affect pigmentation.

The only significant signal that was identified in comparing the pink class with the grey class was observed on BTA3: SNP rs110643545 (ARS‐BFGL‐NGS‐38423) at position 12671675 bp (Fig. 3f; Tables 2 & S1). This SNP is an intronic variant of the *Fc receptor like 3* (*FCRL3*) gene and marks a region that also includes two genes [(*Fc receptor like 1* (*FCRL1*) and *CD5 molecule like* (*CD5L*); positioned at nucleotides 12577541–12610184 and 12508921–12526879 respectively] that have been associated with iris heterochromicity in humans (Jonnalagadda *et al*. 2019).

#### Piebaldism

Contrasting the genotyping data obtained for the animals with solid coat colour and animals with spotted patterns, both single‐marker and haplotype association analyses highlighted a significant peak of association on BTA6 (Table 2, Fig. 4a, Table S1), encompassing the *v‐kit Hardy‐Zuckerman 4 feline sarcoma viral oncogene homolog* (*KIT*) gene, which has already been described to be involved in spotted patterns in cattle (Fontanesi *et al*. 2010a, 2010b; Hayes *et al*. 2010; Jivanji *et al*. 2019).

The haplotype association analysis identified another 22 significant genome regions, only one of which (on BTA5) was also detected by single‐marker analysis (Tables 2 & S2). These regions were in 12 chromosomes (two on BTA1, one in BTA2, two in BTA3, three on BTA5, three on BTA6, one on BTA7, two on BTA10, two on BTA12, two on BTA13, two on BTA20, one on BTA22 and one on BTA29; Tables 2 & S2). Among these regions we could identify a few potential candidate genes that might contribute to the spotted phenotype. The BTA6 region (Table 2) spanning from 87.35 to 87.50 Mb contains the *ADAM metallopeptidase with thrombospondin type 1 motif 3* (*ADAMTS3*) gene (position: 87427184–87706738 bp). Members of the ADAMTS protein family participate in diverse morphogenetic processes during embryonic development and connective tissue maintenance, and a few of them have been involved in melanocyte development and melanoblast migration (Rao *et al*. 2003; Kelwick *et al*. 2015; Tharmarajah *et al*. 2018). The other BTA6 region (spanning from 93.05 to 93.15 Mb) includes the *fraser extracellular matrix complex subunit 1* (*FRAS1*) gene (position: 92951310–93291875 bp). FRAS1 is an extracellular matrix protein, which is involved in regulating membrane adhesion in the epidermal basement during embryonic development (Short *et al*. 2007; Petrou *et al*. 2008). One of the two regions detected on BTA20 by overlapping highly significant or suggestively significant haplotype windows spanning from 2.6 to 2.9 Mb includes the *RAN binding protein 17* (*RANBP17*) gene (position: 2765576–3145422 bp) which encodes a member of the importin‐*β* superfamily of nuclear transport receptors that are involved in diverse biological processes including cell growth, differentiation and development (Koch *et al*. 2000; Kutay *et al*. 2000). The region identified on BTA22 (19.45–19.55 Mb) encompasses the *glutamate metabotropic receptor 7* (*GRM7*) gene (position: 18673820–19567204 bp) that was reported to be a candidate gene for pigmentation in humans (Ganesan *et al*. 2008). Another candidate gene, positioned in the significant region on BTA29 (31.9–32.0 Mb), is the *ETS proto‐oncogene 1*, *transcription factor* (*ETS1*), which encodes an interacting transcription factor involved in melanocyte development (Saldana‐Caboverde *et al*. 2015).

#### Stature

**Figure 5:**
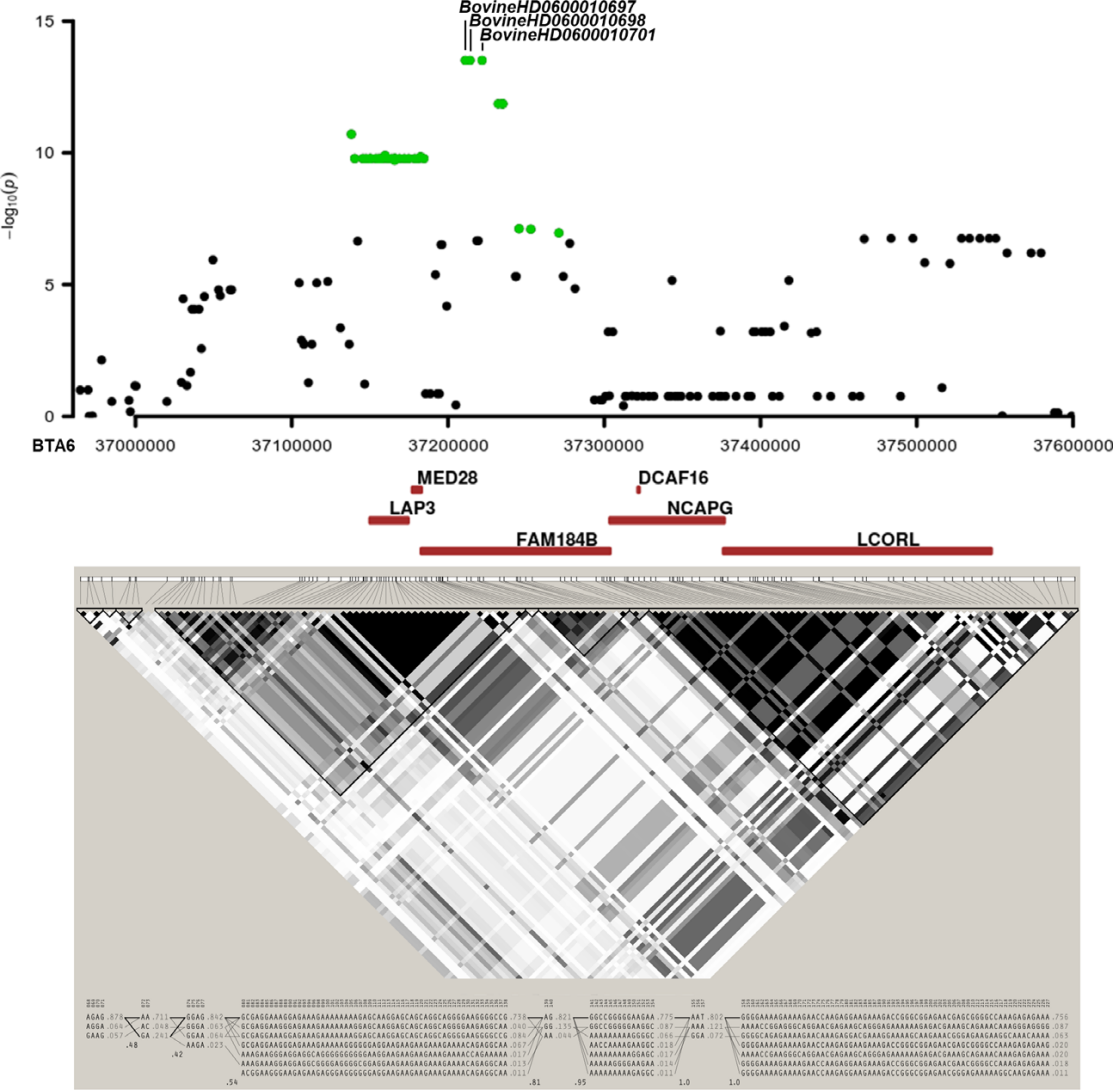
Regional association plot (single‐marker analysis) for the BTA6:37.0–37.6 Mb genomic region associated with stature. Each dot represents an SNP. Significant associations (Bonferroni correction; *α* = 0.05) are highlighted in green. Pairwise LD analysis (haploview tool) of the region, including estimated haploblocks and haplotypes, is reported. LD was measured as *r* ^2^ and it is presented in each box coloured considering the magnitude of linkage.

Only one significant peak, located on BTA6, concordant with both single‐marker and haplotype‐based analyses, was detected (Fig. 4b; Tables 2 & S1). The significant haplotypes spanned a region from 37.10 to 38.00 Mb, which includes the *non‐SMC condensin I complex subunit G* (*NCAPG*) and the *ligand dependent nuclear receptor corepressor like* (*LCORL*) genes (positions: 37277323–37378124 and 37380296–37557106 bp respectively). These genes have already been reported to be associated with stature and body size in cattle and several other species (e.g. Pryce *et al*. 2011; Bouwman *et al*. 2018). The most significant 30 SNPs (*P* from 3.09 × 10^−14^ to 1.91 × 10^−10^) were, however, located upstream of these genes, within the close *family with sequence similarity 184 member B* (*FAM184B*) and *leucine aminopeptidase 3* (*LAP3*) genes (positions: 37181075–37301540 bp and 37140752–37166191 bp, respectively). Figure 5 reports a detailed analysis of this significant region and the related inferred haploblocks, which indicates the presence of two main groups of SNPs in high LD.

A minor peak of suggestive association detected only in the single‐marker analysis (Tables 2 & S1) was identified on BTA14, in the region of the *pleomorphic adenoma gene 1* (*PLAG1*) and *Coiled‐Coil–Helix–Coiled‐Coil–Helix Domain Containing 7* (*CHCHD7*) genes, with the most significant SNP (BovineHD1400007267, rs133704402; *P* = 5.57 × 10^−6^) located at position 23.3 Mb. Inclusion of the significant BTA6 SNPs in the mixed model nullified its effect and did not disclose any other important genomic regions. Other suggestively associated haplotypes (Tables 2 & S2) were identified on BTA16:26.20–26.35 Mb (*P* = 1.40 × 10^−7^), in the *dispatched RND transporter family member 1* (*DISP1*) gene region and on BTA3:105.85–106.00 Mb (*P* = 4.56 × 10^−6^) in the *RLF zinc finger* (*RLF*) gene region, which have both been associated with human height (Kichaev *et al*. 2019).

#### Supernumerary teats and teat length

The genome scans for supernumerary teats (absence vs. presence of supernumerary teats) identified only one suggestively significant SNP (rs110407649; BovineHD1000000240) on BTA10 (position: 894156 bp; Fig. 4c; Table S1) when this defect was analysed in a quantitative manner became significant (*P* = 1.26 × 10^−7^; Fig 4d, Table 2). This marker is an intronic polymorphism in the *MCC regulator of WNT signaling pathway* (*MCC*) gene that encodes a tumour suppressor factor which is thought to negatively regulate cell cycle progression. A minor peak of association (but only suggestive; Tables 2 & S1) was identified on BTA17:60.4 Mb (BovineHD1700017901; rs135074300; *P* = 8.27 × 10^−7^) where the *T‐Box Transcription Factor 3* (*TBX3*) and *T‐Box Transcription Factor 5* (*TBX5*) genes are located. A few other suggestively associated haplotypes were identified on BTA3, BTA7 and BTA29 (Table S2).

Only suggestively significant SNPs and haplotypes were identified for teat length. Markers and haplotypes identified a suggestively associated region for this trait on BTA8:106.95–107.55 Mb (Fig 4e; Tables 2 & S1) which includes the *toll like receptor 4* (*TLR4*) gene. Haplotype‐based analyses identified a second close region on BTA8 (109.25–109.35 Mb) and another region on BTA2:69.15–69.35 Mb.

## Discussion

Despite the small population size of the Reggiana cattle, the monitoring of exterior traits revealed some phenotypic variability within this breed for three pigmentation‐related traits (intensity of the classical red coat colour of the breed, muzzle colour and piebaldism), a body structure‐related trait (stature) and two udder morphometric‐related traits (supernumerary teats and teat length). This phenotypic variability, which can in part be genetically explained, might have been originally derived by the ancestral founder population of this breed or could be subsequently acquired over the years through some unrecorded admixture with other cattle populations, mainly in the past decades. This variability was exploited through the first GWASs carried out thus far in this autochthonous cattle breed that included almost two‐thirds of the extant population in terms of number of cows involved.

The GWASs for the pigmentation‐related traits reported interesting results that could fill some gaps in the genetics of coat colour in the cattle. The classical pheomelanic coat colour of the Reggiana cattle was classified into three groups based on subjective evaluation that defined different grades of red: pale or light red, normal red and dark red. In this way it was possible to test different hypotheses that genetically dissected the trait. Two main groups of factors could be evidenced: one that tends to darken the pheomelanic colouration and another that tends to dilute the classical *fromentino* coat colour. As far as we know, the darkening factors acting on a recessive red background (determined by the epistatic action of the *Extension* locus allele) have not been explored in detail in cattle, probably owing to the low phenotypic variability present in other red cattle populations and breeds and the difficulties in establishing different classes from the basic colour. The subjective approach that we used, which relied on trained technical personnel, was however effective in defining different groups of animals, as demonstrated by the results obtained from the GWAS that relied on the phenotypes retrieved in this way. The main significant peak that we identified in the analyses that compared the dark animals with all other cows pointed out *EDN3* as the candidate gene involved in the darkening of red coat colour. Several *in vitro* studies using quail and mouse embryos have reported that *EDN3* affects the melanocyte lineage population by increasing their number in a dose‐dependent manner (Lahav *et al*. 1996, 1998; Reid *et al*. 1996; Opdecamp *et al*. 1998; Dupin *et al*. 2000). In addition, exogenous overexpression of *EDN3* driven by keratin 5 in transgenic mouse embryos can induce proliferation of melanocyte precursors and leads to hyperpigmentation on most areas of the skin (Garcia *et al*. 2008). Dermal hyperpigmentation in Silkie chicken (*Fibromelanosis* locus, *Fm*; Bateson & Punnett 1911; Hutt 1949) is caused by an inverted duplication and junction of two genomic regions, one of which contains the *EDN3* gene (Dorshorst *et al*. 2011). Developing Silkie embryos during the time in which melanoblasts are migrating have increased *EDN3* expression that is maintained in the adult chicken skin tissue (Dorshorst *et al*. 2011). These reports in other species also suggest that variability in the cattle *EDN3* gene might be the main determinant of the observed darker red colour of the coat in some Reggiana cattle. We could also propose that a darkening coat colour locus may exist in cattle and this is homologous to the *Fibromelanosis* locus reported in chicken. It will be interesting to evaluate if skin tissues of cattle of different red intensity have different levels of expression of this gene and establish the precise molecular mechanisms by which *EDN3* determines this peculiar effect of coat colour in cattle and its interactions with the Extension locus.

Other genetic factors are also expected to contribute to darkening the red colour as an additional nine significant regions that are associated with a dark red colour were identified, a few of which contain candidate genes already known to be involved in some processes related to pigmentation. For example, another region on BTA5 contains *WIF1*, which is constitutively expressed in melanocytes, where it promotes melanogenesis (Park *et al*. 2014).

The opposite phenotypic effect, i.e. dilution or lightening of the standard coat colour, was mainly associated with some other genomic regions. The most important one includes the *PMEL* gene (ENSBTAT00000005250.5 and ENSBTAP00000005250.4 codes hereafter used for the description of variations) that is responsible for the *Dilution* coat colour locus, already described in some breeds by classical genetic studies and molecular investigations, which have reported a few mutated dominant or partially dominant alleles over the wt allele at this main locus (Searle 1968; Olson 1999; Ruvinski 2014). These mutated alleles, only partially characterised at the molecular level, have been referred to according to the breed in which they were first investigated and detected: *D^C^* (Charolais type), caused by the *PMEL* missense mutation c.64G>A (rs71855305; p.Gly22Arg) reported by several studies (Oulmouden *et al*. 2005; Gutiérrez‐Gil *et al*. 2007; Kühn & Weikard 2007); *D^S^* (Simmental/Hereford type), partially associated with a congenital hypotrichosis in cattle (commonly referred to as rat‐tail syndrome) and suggested to be caused by the combination of two *PMEL* mutations – the three base deletion in exon 1, p.Leu19del (rs385468954), and another missense mutation at codon 612, p.A612E (rs378894329; Hecht & Campbell 2005; Hecht 2006); and *D^H^* (Highland/Galloway type), reported to have the p.Leu19del but partially characterised for other mutations (Schmutz & Dreger 2013). The genotyping data we obtained in Reggiana excluded the presence of the *D^C^* allele in the breed as all animals were homozygous for G at the *PMEL* c.64G>A SNP. A few Reggiana cattle carried the p.Leu19del mutation (allele *D^S^* or *D^H/G^*), always in a heterozygous condition. The p.Leu19del allele could have been introgressed into Reggiana through crossbreeding with Simmental cattle, which probably happened in the past. In the Reggiana breed, however, no reports have suggested the presence of the rat‐tail defect that, however, might not be completely associated with mutations in the *PMEL* gene (Knaust *et al*. 2016). The frequency of the p.Leu19del allele in the Reggiana population was 2.1% which, compared with the frequency of the diluted cattle identified phenotypically (5.6%), might indirectly suggest that in Reggiana the mutated allele could be dominant or partially dominant. However, a formal analysis would be needed to establish its precise mode of inheritance. In addition, it also seems clear that other genetic factors are involved in determining a diluted coat colour in the Reggiana breed because the GWASs reported a few other significant genome regions, the most relevant of which included the *ADGRV1* gene. In humans, mutations in this gene cause Usher syndrome type IIC (Weston *et al*. 2004), which is characterised by hearing deficiencies and progressive development of retinitis pigmentosa. Markers in this gene have been also reported to be highly associated with blonde hair in a large case–control study against brown and black hair in European human populations (Morgan *et al*. 2018; Kichaev *et al*. 2019).

The pink or pale muzzle (the standard muzzle colour reported by the Reggiana Herd Book) was clearly associated with the *MC1R e* allele. The presence of a black muzzle was largely explained by non *e* allele(s), referred to with the symbol *E*, segregating in the breed population. The phenotypic expression of *E* allele(s) might be only evident at the muzzle colour level as, at the coat level, *E* might confer a reddish/brownish colouration that could be similar to the classical *fromentino* red colour of the breed. The effect of *E* allele(s) could be modified by other loci, as described above, that might determine the observed grades of red, confusing the phenotypic assignment at different *Extension* genotypes. The three coat colour classes discussed above, however, were not associated with *MC1R* variability, as no significant signals were reported on BTA18 in the GWASs that were carried out for coat colour intensity. It will be interesting to evaluate which of the other *MC1R* alleles already described in brown/red cattle breeds (*E*, *E^1^* or *e^f^*; Rouzaud *et al*. 2000; Graphodatskaya *et al*. 2002; Lee *et al*. 2002) are present in Reggiana and their potential effects on this peculiar phenotype. We can, however, exclude *E^D^*, which would behave as dominant black allele on coat colour, as already demonstrated in crossbred Holstein × Reggiana cattle (ANABORARE, unpublished observation). Despite the large effect of the *MC1R* gene, other genetic factors are involved in determining the black muzzle colour in Reggiana cattle as significant SNPs and haplotypes have been identified in another 11 genome regions. The intermediate muzzle colour class (grey) appeared to be determined by other genetic mechanisms, separated by those determining the black muzzle colour. Only one significant region on BTA3 was detected in the GWASs that compared pink vs. grey muzzle groups of cattle. Additional studies with a larger number of animals would be needed to complete the dissection of this pigmentation‐related trait that appears to be more complex than expected.

The third pigmentation trait that was investigated in this study was mainly associated with variability at the *KIT* gene, which is a well‐known determinant of piebaldism in cattle (Fontanesi *et al*. 2010a, 2010b; Hayes *et al*. 2010; Jivanji *et al*. 2019). Markers at this gene were the most significant ones even if the multifactorial component determining piebaldism (Hayes *et al*. 2010; Jivanji *et al*. 2019) was confirmed by several significant SNPs and haplotypes in other genome regions. In most cases, these significant additional regions harboured potential candidate genes (i.e. *ADAMTS3*, *ETS1*, *FRAS1*, *GRM7* and *RANBP17*) whose functions could be directly or indirectly involved in the migration and development processes of melanoblasts and melanocytes, which are absent in the white regions of the body. Spotted cattle emerge sporadically in Reggiana from sires and dams that had a solid red coat colour without any spotted patterns, suggesting that piebaldism might be a quantitative trait determined by several genetic factors in combination, most of which are recessive (Olson, 1999; Hayes *et al*. 2010).

Another classical quantitative trait that we explored in Reggiana cattle was the stature of the animals. This trait has already been investigated in other cattle populations by some studies that identified the major genetic components involved. All reports are concordant in identifying the most relevant region located on BTA14, encompassing the *PLAG1/CHCHD7* genes, followed by several other major QTL located in other chromosomes, including the region of the *LCORL/NCAPG* genes on BTA6 (e.g. Pryce *et al*. 2011; Bolormaa *et al*. 2014; Randhawa *et al*. 2015; Bouwman *et al*. 2018). The results we obtained in Reggiana indicated that the highest genetic contribution on this trait derived from the same region on BTA6 but SNPs on BTA14 were only suggestively significant. As identified in other cattle breeds and populations, this region on BTA6 has been also reported to have pleiotropic effects on related traits (feed intake, growth, carcass and meat production traits, birth weight, body structural traits; e.g. Nkrumah *et al*. 2007; Eberlein *et al*. 2009; Gutiérrez‐Gil *et al*. 2009; Setoguchi *et al*. 2009; Lindholm‐Perry *et al*. 2011; Sahana *et al*. 2015; Xia *et al*. 2017). Even if additional phenotypes were not available in Reggiana, the large effects observed in this Italian local breed could be indirectly due to the fact that its dual‐purpose structure is still largely evident in the genome of most Reggiana cattle as also inferred by the results of a study of signatures of selection (Bertolini *et al*. 2020). It is also worth noting that most significant BTA6 SNPs in Reggiana were upstream of the *LCORL* gene (Fig. 5) as already reported in another study (Purfield *et al*. 2019). The haplotype structure of this region indicated the presence of two major haplotype blocks that in combination with that mentioned above might suggest that the close gene *FAM184B* could play a relevant role on this trait or that regulatory variants affecting the expression of the downstream genes *LCORL/NCAPG* might be involved. The complexity and the general high LD level in this region in many cattle populations have prevented to clarify thus far which are the genetic mechanisms or genes that are involved in determining body structural differences (Bouwman *et al*. 2018; Purfield *et al*. 2019).

Owing to the relevance of the udder in dairy cattle, a large number of studies have already reported QTL that explain, at least in part, the genetic variability for udder traits and defects. Supernumerary teats (indicated also as hyperthelia) are any abnormal teats in addition to the usual four teats that correspond to the four usual functional mammary glands. Supernumerary teats are an undesired and heritable trait in cattle (Brka *et al*. 2000, 2002; Pausch *et al*. 2012; Joerg *et al*. 2014). It is considered a relevant defect as it may cause disturbances of machine milking, and because if it is combined with supernumerary mammary glands, it may increase udder health problems (Skjervold 1960; Wiener 1962; Brka *et al*. 2000, 2002). About 31% of the Reggiana cows had at least one supernumerary teat. This frequency is similar to that reported in other breeds. For example, Brka *et al*. (2002) observed that the frequencies of affected German Brown Swiss and German Simmental were 31.2% and 44.3% respectively. The polygenic determinism and, possibly, breed‐specific components affecting this defective trait have also been demonstrated by the partial overlapping of the results across the GWASs carried out thus far in cattle (Pausch *et al*. 2012; Joerg *et al*. 2014; Butty *et al*. 2017). Two of these studies are consistent in indicating a major QTL in the region of BTA17 containing *TBX3* and *TBX5* genes (Pausch *et al*. 2012; Butty *et al*. 2017). This region was also suggestively significant in our GWASs in Reggiana cattle. *TBX3* and *TBX5*, originating from gene duplication, belong to the T‐box family of genes which encodes transcription factors that play an important role in development programmes of many animal species. Mutations in the human *TBX3* gene causes ulnar–mammary syndrome, a pleiotropic dominant disorder characterised by ulnar defects, mammary and apocrine gland hypoplasia and genital abnormalities (Bamshad *et al*. 1997). TBX3 is a downstream target of the canonical Wnt pathway involved in mammary gland initiation in mouse embryos (Eblaghie *et al*. 2004; Renard *et al*. 2007). The *MCC* candidate gene that we identified in the significant region detected in Reggiana on BTA10 is also involved in the non‐canonical Wnt signalling pathway (Renard *et al*. 2007; Young *et al*. 2014). In addition, the main QTL for supernumerary teats reported by Buffy *et al*. (2017) on BTA5 includes another gene involved in the Wnt signalling pathway (*leucine rich repeat containing G protein‐coupled receptor 5*, *LGR5*; Plaks *et al*. 2013). All of these elements seem to show that in cattle, alterations of the Wnt signalling pathway could represent one of the basic biological mechanisms that determine this defect.

Only a few suggestively significant markers were associated with teat length, despite the moderate estimated genomic heritability (0.35), which was similar to that of other investigated traits in this study. This might suggest that no major genes affect this quantitative trait, as it is also possible to deduce from the several previous studies that investigated this trait in other cattle breeds (summarised in Hu *et al*. 2019).

The two teat‐related traits investigated in this study are directly or indirectly involved in several aspects associated with milking ability. By interfering with the correct placement of the conventional milking machine, they reduce judgement scores at exhibitions and thus decrease the economic value of the animals. They also present risk factors for mastitis and reduced welfare of the cows, considering the practice of surgical elimination of supernumerary teats (Steiger & Grünenfelder 1988; Butty *et al*. 2017). Therefore, their genetic dissection should be further strengthened, increasing the number of investigated animals to cover the whole Reggiana population in order to obtain meaningful results.

## Conclusions

This study demonstrated that, by exploiting variability within an autochthonous cattle genetic resource, it was possible to obtain interesting results on associated markers that contributed to clarifying the genetic mechanisms of several exterior traits in cattle. It will be useful to explore variability in the *EDN3* gene and identify the causative mutation(s) of this coat colour darkening locus in Reggiana and eventually in other cattle breeds. Some of the results on coat colour and muzzle colour can be directly implemented to select against undesired phenotypes that are not allowed by the Herd Book standard of the Reggiana breed. Other results that might need to be confirmed could be useful to start selection programmes to improve udder conformation and related milking performances and redefine the body structure of the Reggiana animals.

## Conflict of interests

The authors declare they do not have any conflict of interests.

## Supporting information


**Figure S1** Q–Q plots of the single‐marker GWASs carried out in the Reggiana cattle breed.
**Figure S2** Q–Q plots of the haplotype‐based GWASs carried out in the Reggiana cattle breed.

**Table S1** SNPs with *P* < 5.00 × 10^−6^ in GWASs for the investigated traits and defects in the Reggiana cattle breed.

**Table S2** Haplotypes with *P* < 5.00 × 10^−6^ in GWASs for the investigated traits and defects in the Reggiana cattle breed.

## Data Availability

The datasets used and analysed during the current study are available from the corresponding author on reasonable request and can be shared after an agreement on their use with University of Bologna and ANABORARE.
