# Supplementary material for: Exploiting within‐breed variability in the autochthonous Reggiana breed identified several candidate genes affecting pigmentation‐related traits, stature and udder defects in cattle
Source: Anim Genet. 2021 Jun 28;52(5):579–97. doi: 10.1111/age.13109 (PMC8519023; doi:10.1111/age.13109)

**Supplementary material**

**Exploiting within breed variability in the autochthonous** **Reggiana breed identified several candidate genes affecting pigmentation-related traits, stature and udder defects in cattle**

Samuele Bovo, Giuseppina Schiavo, Hamed Kazemi, Giulia Moscatelli, Anisa Ribani, Mohamad Ballan, Massimo Bonacini, Marco Prandi, Stefania Dall’Olio and Luca Fontanesi

**Table S1.** Single nucleotide polymorphisms presenting a *P* < 5.00×10^-06^ in GWA analyses for the morphological traits and defects in the Reggiana cattle breed. Results are sorted by *P* of association.

**[Results are provided in the attached spreadsheet]**

**Table S2.** Haplotypes presenting a *P* < 5.00×10^-06^ in GWA analyses for the morphological traits and defects in the Reggiana cattle breed. Results are sorted by *P* of association.

**[Results are provided in the attached spreadsheet]**

**Figure S1.** Quantile-quantile plots of the single-marker genome-wide association studies carried out in the Reggiana cattle breed. Inflation factor (λ_GC_) is reported.


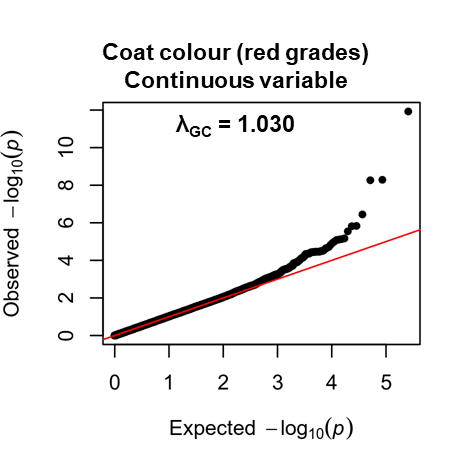

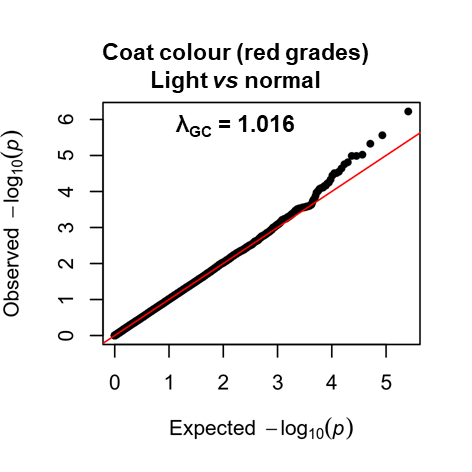

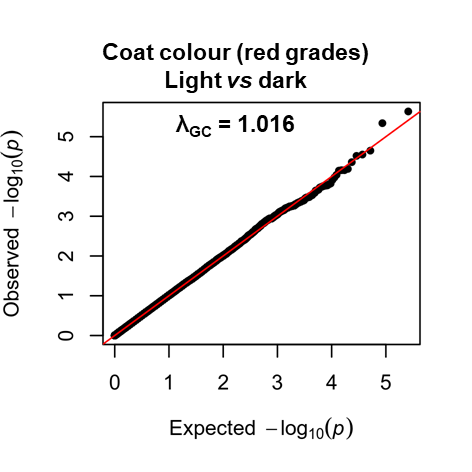

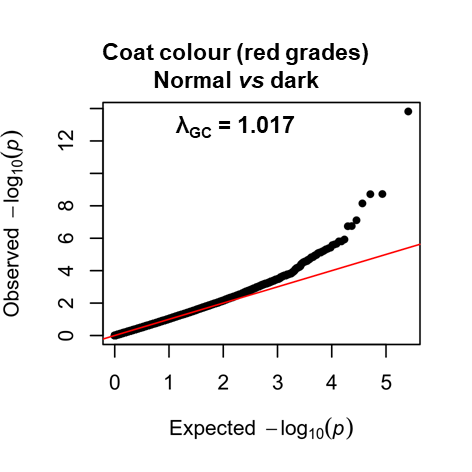

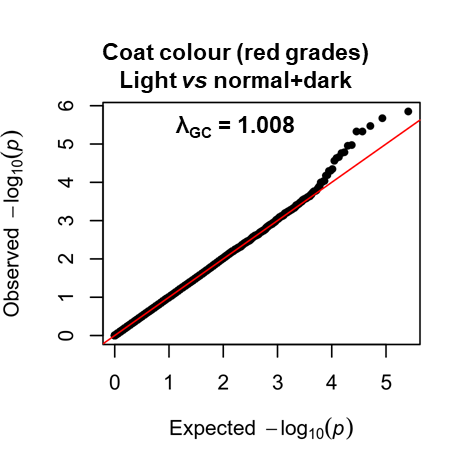

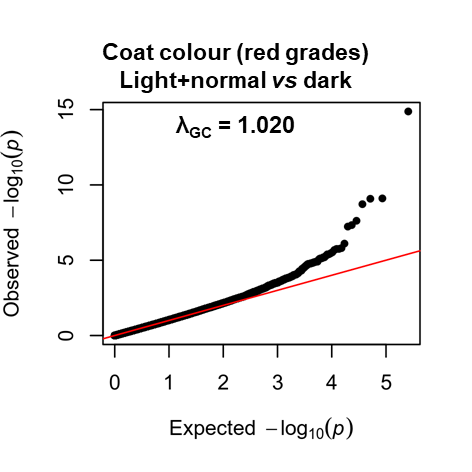

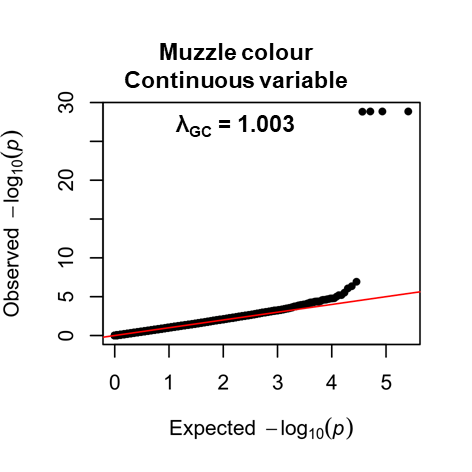

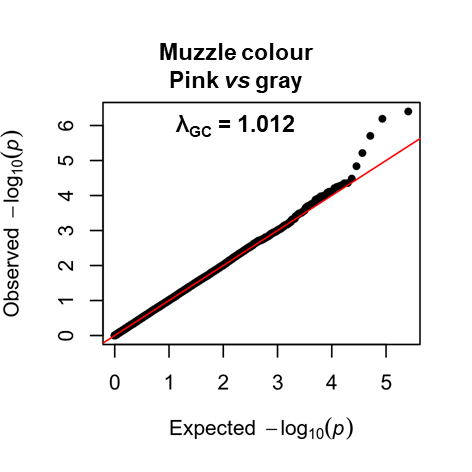

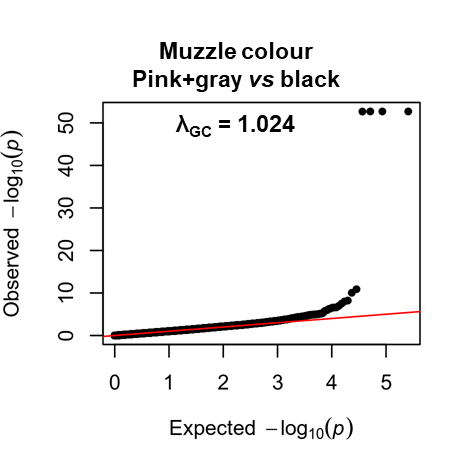

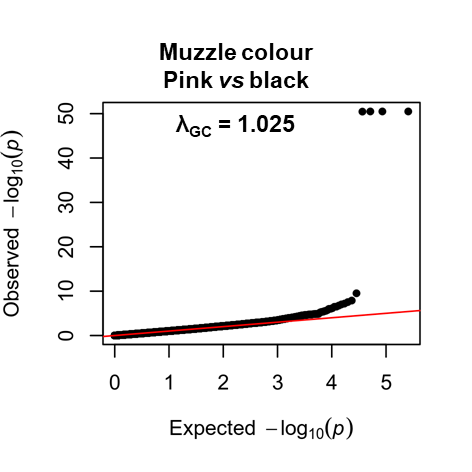

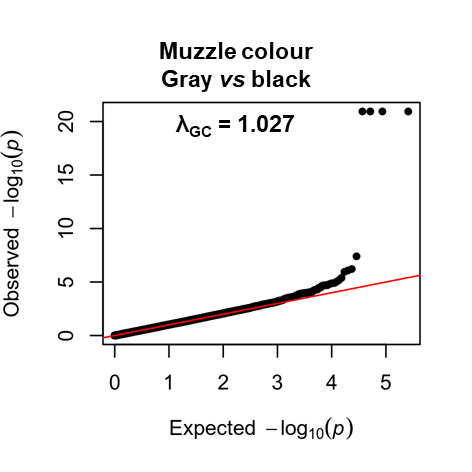

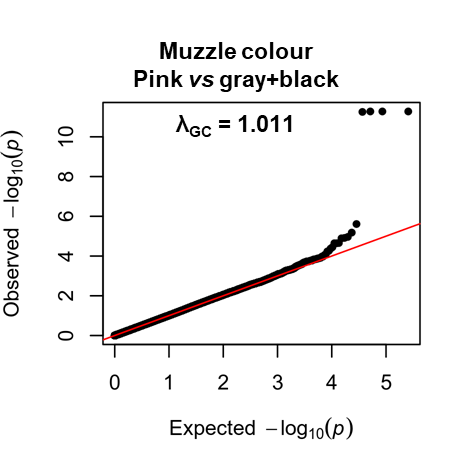

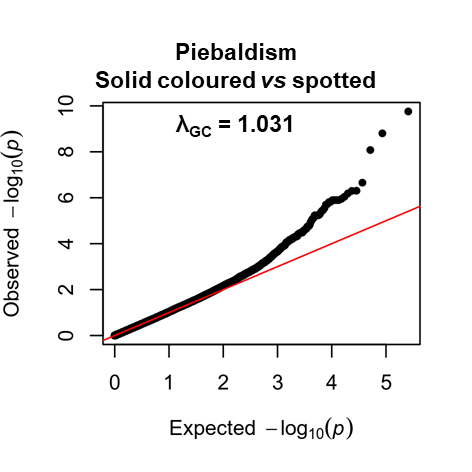

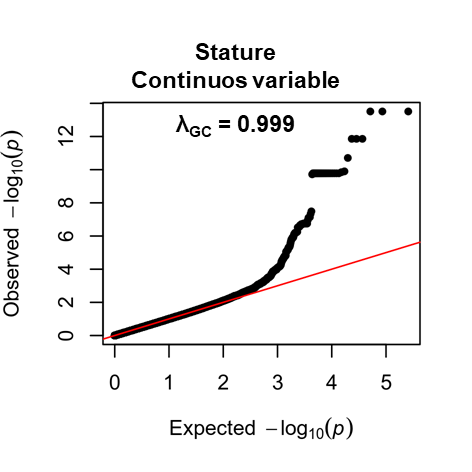

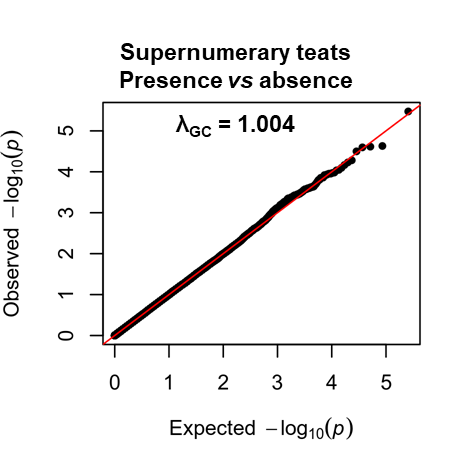

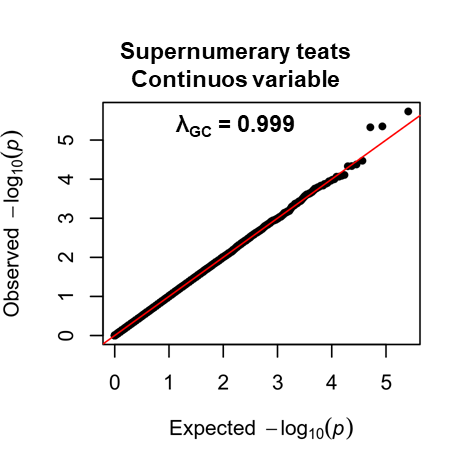

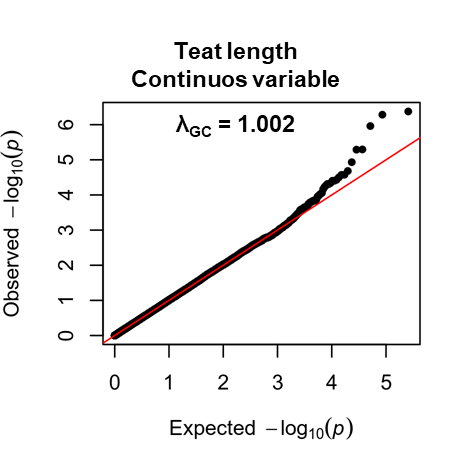


**Figure S2**. Quantile-quantile plots of the haplotype-based genome-wide association studies carried out in the Reggiana cattle breed. Inflation factor (λ_GC_) is reported.


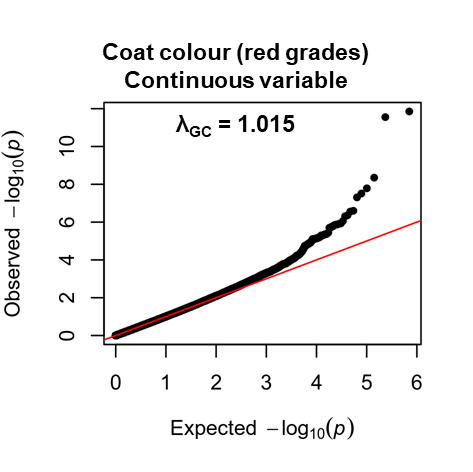

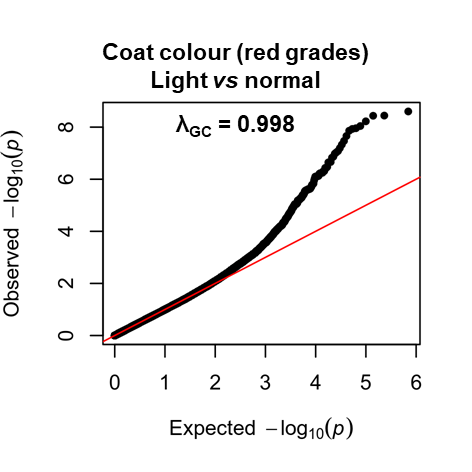

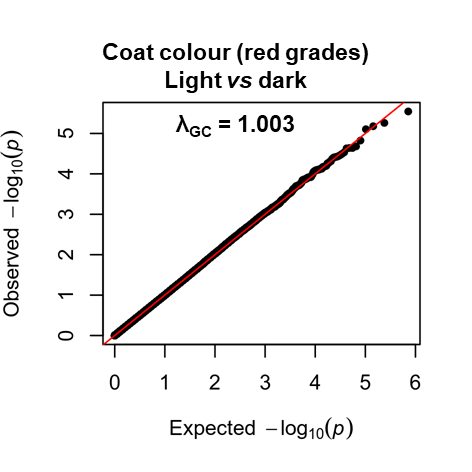

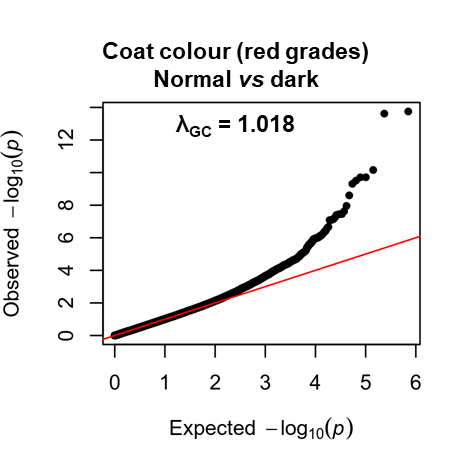

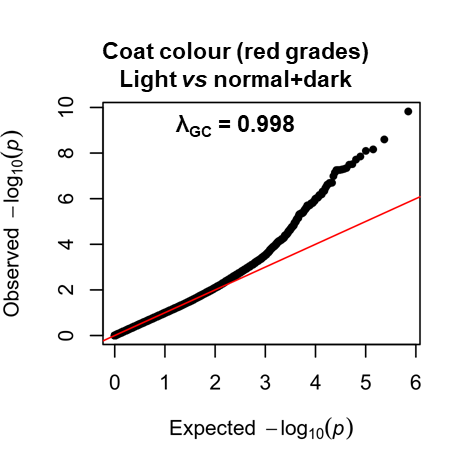

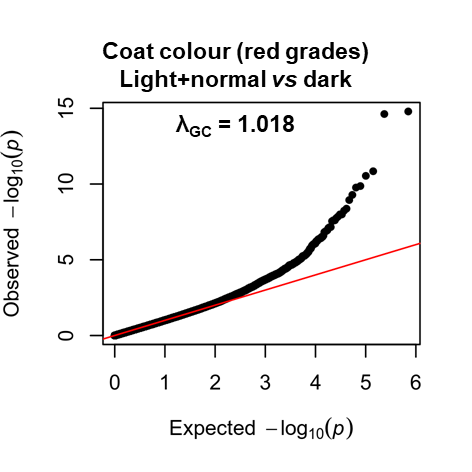

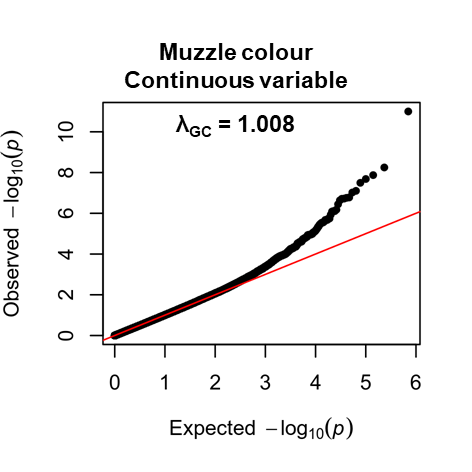

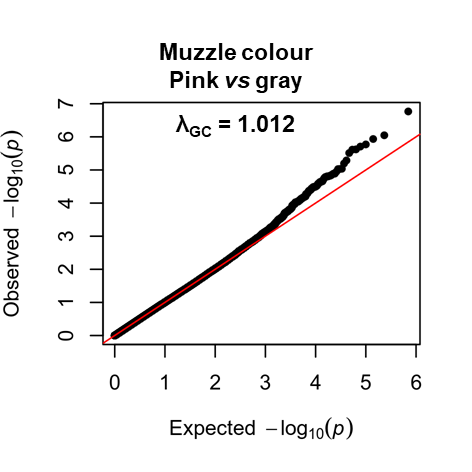

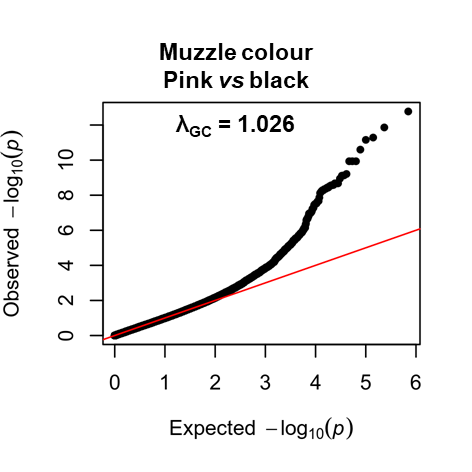

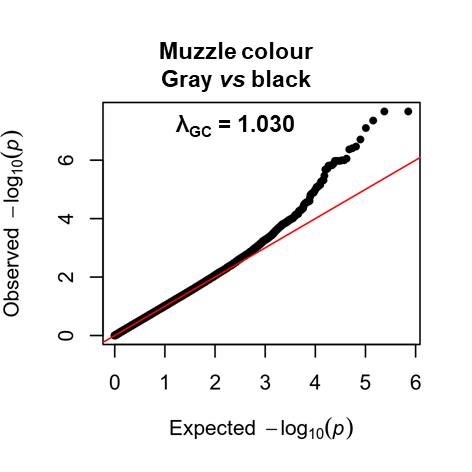

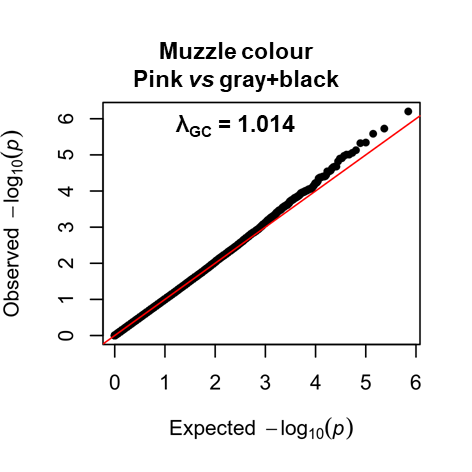

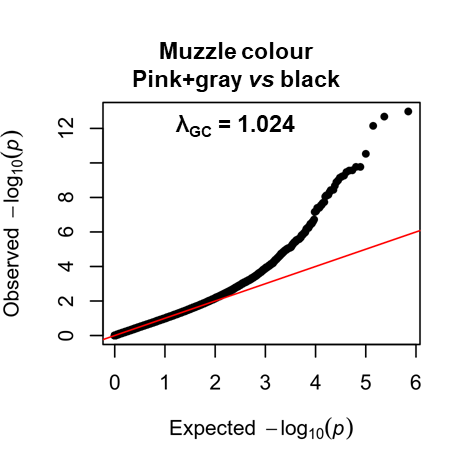

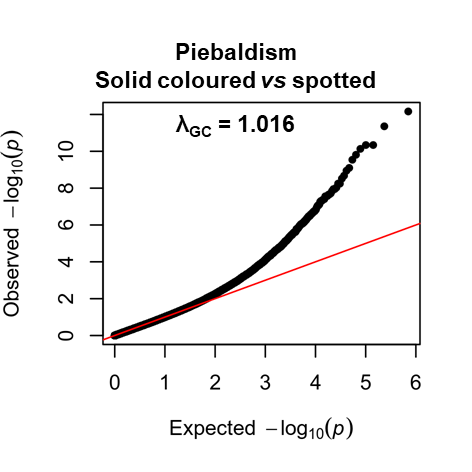

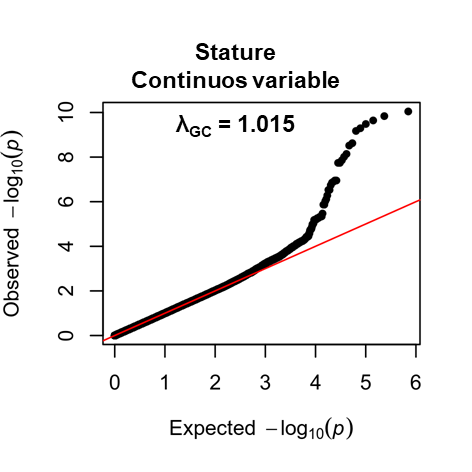

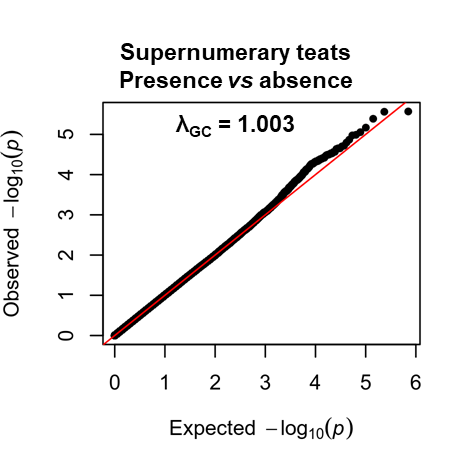

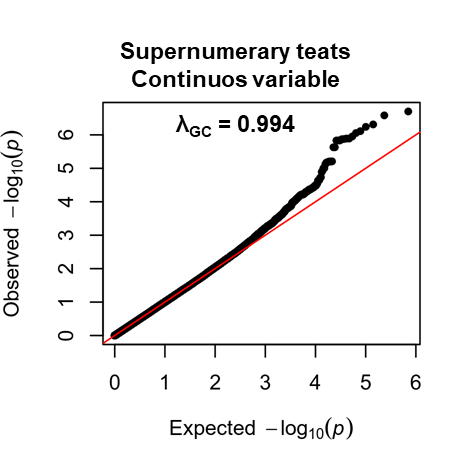

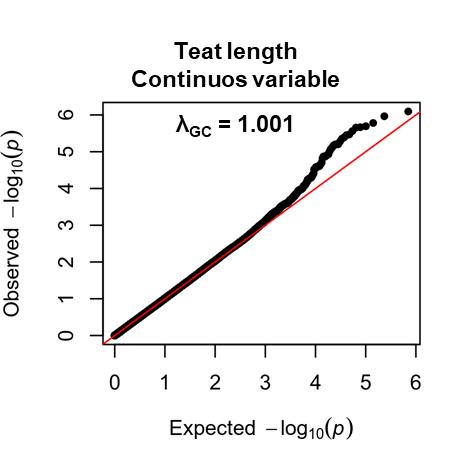

Supplement: Supplementary file 1 — Figure S1 Q–Q plots of the single‐marker GWASs carried out in the Reggiana cattle breed. Figure S2 Q–Q plots of the haplotype‐based GWASs carried out in the Reggiana cattle breed. [file AGE-52-579-s001.docx]
